# Attitudes Toward Interprofessional Education Among Medical Students of a German Private Medical School Network

**DOI:** 10.1007/s40670-026-02712-9

**Published:** 2026-03-27

**Authors:** Holger Joswig, Urs Lichtenauer, Ilona Renken-Olthoff, Olaf Schenk, Britta Wulfhorst

**Affiliations:** 1 Medical School Hamburg, University of Applied Sciences and Medical University, Hamburg, Germany; 2 Health and Medical University, Olympischer Weg 1, Potsdam, 14471 Germany; 3 Medical School Berlin, Berlin, Germany; 4 Health and Medical University Erfurt, Erfurt, Germany; 5 Institute of Research and Education, Hamburg, Germany

**Keywords:** Education, Germany, Interprofessional, Medical school, Private university

## Abstract

**Background:**

Interprofessional education (IPE) in healthcare remains insufficiently studied, despite the World Health Organization's strong emphasis. Research on attitudes toward IPE among medical students (MS) from private medical schools is particularly limited.

**Objective:**

To study the attitudes toward IPE among MS from German private medical schools.

**Methods:**

Using an online survey, positive and negative predictors of IPE attitudes, as measured by the validated German version of the Interprofessional Learning Scale of the University of the West of England Interprofessional Questionnaire (UWE-IP-D ILS), were identified from 256 MS at four private German medical schools. Additionally, MS selected preferred preclinical / clinical curriculum subjects for IPE.

**Results:**

The overall UWE-IP-D ILS mean (standard error) score was 20.27 (± 8.16). Multiple regression analysis identified female sex as a significant positive predictor of positive IPE attitudes, even after Bonferroni correction (*p* = 0.006). However, IPE attitudes tended to decrease over the study years (non-statistically significant trend). Basic sciences, mental health disciplines, and family medicine were most frequently identified as most appropriate subjects for IPE. More than 80% of participants expressed willingness to allocate teaching activities to IPE within and outside the regular curriculum.

**Conclusions:**

The survey results support the implementation of structured IPE programs and guide IPE embedding in the German medical curriculum.

**Supplementary Information:**

The online version contains supplementary material available at 10.1007/s40670-026-02712-9.

## Introduction

German medical education is historically and uniquely structured into a 2-year preclinical and a 4-year clinical segment followed by a so-called practical year in hospital. With its focus on medicine-related scientific classes, the preclinical segment is comparable to the 4-year bachelor’s degree that serves as a requirement to apply for medical school in the United States. Medical school matching in Germany is operated by one central governmental nonprofit trust and heavily reliant on the Abitur (German high school equivalent) grade point average [1]. Private medical schools, albeit under German jurisdiction, are not bound to the aforementioned institution and therefore free in their selection of medical students (MS) by own criteria. Otherwise, the medical curricula of both tiers are indistinct from each other with all MS subject to two national state board exams [2]. The IRO group is a network of private universities that includes four private medical schools, Medical School Hamburg – University of Applied Sciences and Medical University (MSH), Medical School Berlin (MSB), Health and Medical University (HMU) Potsdam and HMU Erfurt. Interprofessionality in teaching and interdisciplinarity in research form a core pillar of the IRO group’s philosophy. Interprofessional collaboration in healthcare improves patient safety and outcomes while also enhancing satisfaction among patients and healthcare workers [3]. *“Interprofessional education (IPE) occurs when two or more professions learn about*,* from and with each other to enable effective collaboration and improve health outcomes”* [4], which is key to later interprofessional practice [5]. As such IPE was anchored in the upcoming legal regulatory framework of the German medical curriculum [6], which mandates IPE competencies requiring future physicians to master collaboration with other doctors and health professions—explicitly elevating interprofessional teamwork. In their scoping review, Grace et al. [7] found that international health education institutions from North America, Europe, Australia, South Africa, Singapore and Saudi Arabia had partially (28%) or wholly (72%) integrated IPE in their curriculum. Likewise, for dentistry multiple surveys indicated that the majority of dental schools in the United States and Canada had IPE integrated into their curricula [8]. A significant increase in positive attitudes toward IPE following learning-interventions was found in the review by Berger-Estilita et al. [9] where most of the studies were from the United States and used the Readiness for Interprofessional Learning Scale (RIPLS). A few authors previously looked at attitudes toward IPE among MS in German-speaking countries [10–14]. Juschka et al. [13] assessed the status quo of IPE courses for midwifery and MS but did not apply validated measures. In order to explore the perceptions of interdisciplinary knowledge, teaching content, interprofessional status of medical and dental professions, and attitude toward IPE of MS and dental students at the Johannes Gutenberg-University of Mainz, Germany, Hackenberg et al. [12] created their own questionnaire. Other authors used the Interdisciplinary Education Perception Scale [14], the RIPLS [11], or the validated German version [15] of the University of the West of England Interprofessional Questionnaire (UWE-IP-D) [10, 16, 17]. However, the aforementioned authors did not consider private medical schools, which represent a blind spot in IPE research thus far.

This survey aims, for the first time, to shed light on what the picture on IPE is in a private medical school. Besides the application of a validated IPE-measurement tool (UWE-IP-D), we sought to better understand IPE attitudes by inquiring if and how many hours of curricular and extra-curricular time the MS would be willing to invest into IPE. Furthermore, modifiers of IPE-attitudes are explored, and the subjects of the German medical curriculum deemed most suitable for IPE are determined to better understand where to embed IPE.

## Materials and Methods

### Survey Conduction

The study protocol was reviewed and approved by the MSH Ethics Committee, approval number MSH-2024/386. An email-invitation for a voluntary and anonymous online survey (Unipark, Tivian) was sent by the Student Affairs Office to all MS of the four medical schools on February 12, 2025. Being rolled in as a MS was the only inclusion criteria, and no exclusion criteria applied. Neither did the study conductors see the email addresses nor did they obtain any other personally identifiable information, and all data was treated confidential. Following a reminder email after one week, the survey closed on March 31, 2025.

### Survey Content

Informed consent was obtained at the start of the online survey, and the World Health Organization’s definition of IPE [4] provided to the participants. The following data was obtained during the questionnaire (see also supplementary file): sex, age, medical school affiliation, study year, and whether MS had previous occupation / vocational training in either health care or outside health care, and/or previous IPE experience.

In order to capture MS’ attitudes toward IPE using a validated instrument, the “Interprofessional Learning Scale (ILS)” of the University of the West of England Interprofessional Questionnaire (UWE-IP) developed by Pollard et al. [16, 17] and translated in German (UWE-IP-D) by Mahler et al. [15] was chosen. The scale consists of 5-point Likert responses ranging from 1 (strongly agree) to 5 (strongly disagree) with overall scores ranging from a minimum of 9 to a maximum of 45 points indicating positive and negative attitudes towards interprofessional learning, respectively. Tailored to the study aim and to keep the overall survey length short and reduce participant attrition, the other three scales, “Communication and Teamwork Scale” (9 items), “Interprofessional Interaction Scale” (9 items), and “Interprofessional Relationships Scale” (8 items) were omitted.

Participants were asked to select the subjects they considered most suitable for IPE (multiple selections allowed). Preclinical students (year 1 and 2) could choose only subjects from the preclinical curriculum, whereas clinical students (beyond year 2) were offered all preclinical and clinical subjects.

At the end of the survey, participants were asked about their willingness to invest extracurricular time (i.e. outside the regular course schedule) in IPE and the number of hours they would commit (0 up to 5 h). Lastly, they indicated the percentage of mandatory teaching activities they believed should be conducted in an IPE setting (0 to 100%).

### Statistical Analysis

**Fig. 1:**
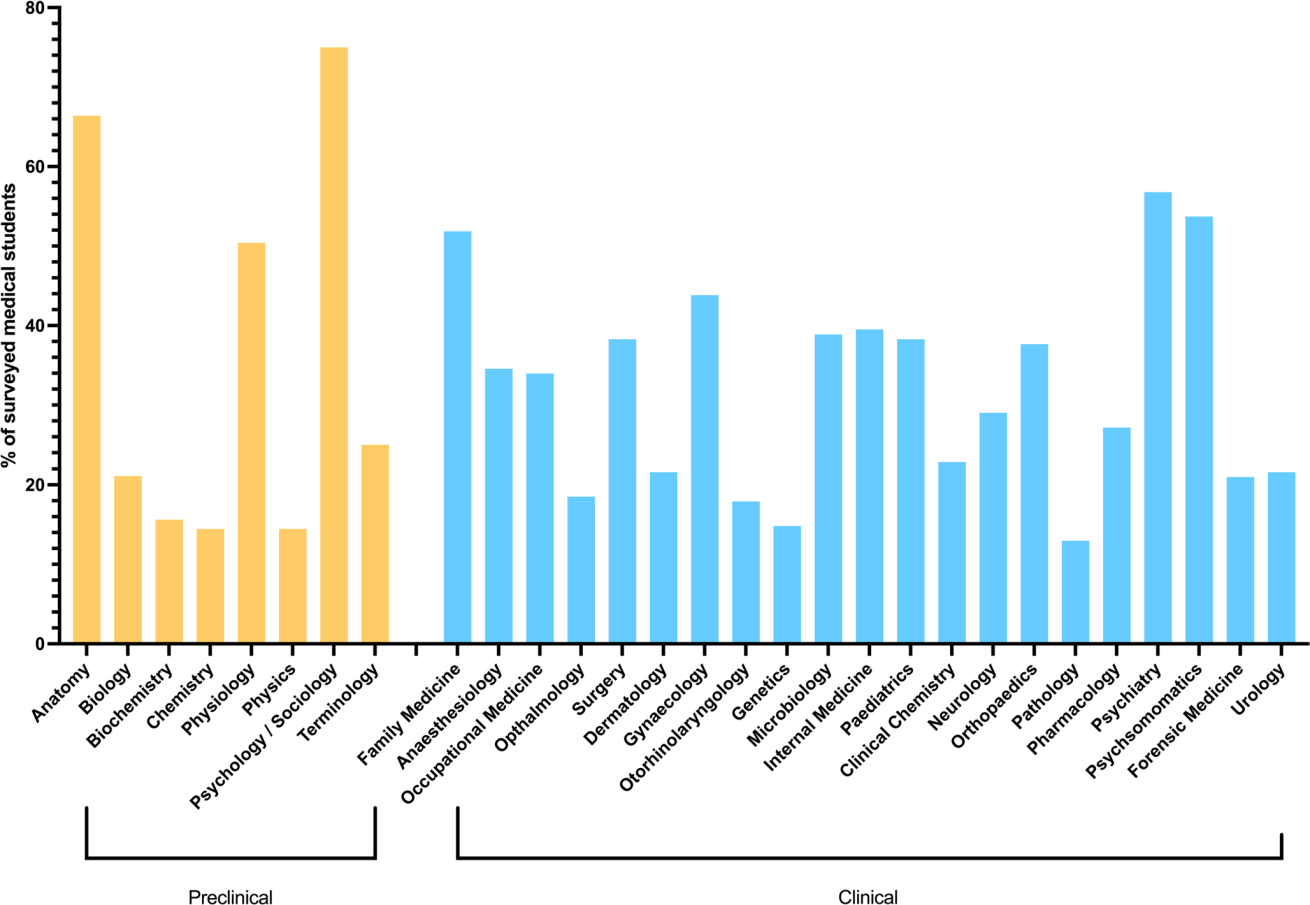
Percentages of subjects deemed most suitable for interprofessional education during the preclinical (left; *n* = 256) and clinical segment (right; *n* = 162) by medical students at a private German medical school network

The internal consistency of the UWE-IP-D ILS was tested with a Cronbach’s alpha analysis. Multiple regression analysis was performed using SPSS IBM Version 30.0.0.0 (172) (IBM Corp.). P-values < 0.05 were considered statistically significant. Percentages of subjects deemed most suitable for IPE were graphically represented in Fig. 1 using Graphpad Prism 9 for macOS, Version 9.5.1.

## Results

Two-hundred-fifty-six MS completed the survey, and the respective medical school’s response rates are given in Table 1. Sex, age, medical school affiliation, study year, previous occupation / vocational training in either health care or outside health care, and/or previous IPE experience, UWE-IP-D ILS overall and per item means and standard deviations, as well as hours and percentage of voluntary IPE are illustrated in Table 2. Cronbach’s alpha for internal consistency of the UWE-IP ILS was 0.938. Overall, 86.7% of survey participants expressed willingness to allocate some portion of all teaching activities to IPE. Additionally, 82.4% would participate in voluntary IPE for at least one hour per month.

**Table 1:** Absolute numbers (percentage) of participating medical students from four private medical schools

Medical school MSH MSB HMU Potsdam HMU Erfurt	256/8131 (3.2%) 108/4321 (2.5%) 15/2320 (0.7%) 121/985 (12.3%) 12/505 (2.4%)

**Table 2:** Characteristics of *n* = 256 medical students participating in a survey on interprofessional education (IPE). SE = standard error. MSB = Medical School Berlin, MSH = Medical School Hamburg, HMU = Health Medical and University, UWE-IP-D ILS = Interprofessional Learning Scale of the University of the West of England Interprofessional Questionnaire (German version)

Sex Male Female	*n* (%) 99 (38.7%) 157 (61.3%)
Age	mean ± SE, range 23.93 ± 3.32, 18–38 years
Medical school MSH MSB HMU Potsdam HMU Erfurt	n (%) 108 (42.2%) 15 (5.9%) 121 (47.3%) 12 (4.7%)
Medical school year 1 2 3 4 5 6	n (%) 108 (42.2%) 54 (21.1%) 37 (14.5%) 33 (12.9%) 24 (9.4%) 0 (0%)
Previous occupation / training in health care outside health care	n (%) 136 (53.1%) 34 (13.3%)
Previous IPE experience	n (%) 124 (48.4%)
UWE-IP-D ILS item 1 item 2 item 3 item 4 item 5 item 6 item 7 item 8 item 9	mean ± SE, range 20.27 ± 8.16, 9–45 2.57 ± 1.17, 1–5 2.22 ± 1.15, 1–5 2.54 ± 1.17, 1–5 1.82 ± 0.96, 1–5 2.57 ± 1.18, 1–5 2.26 ± 1.13, 1–5 1.88 ± 0.97, 1–5 2.28 ± 1.14, 1–5 2.13 ± 1.08, 1–5
% IPE	mean ± SE, range 16.99 ± 16.07, 0–80
Voluntary IPE extra hours	mean ± SE, range 2 ± 1.41, 0–5 h

### Multiple Regression Analysis

Females scored significantly (*p* = 0.006) lower on the UWE-IP-D ILS (i.e. more positive attitudes toward IPE; Table 3), and desired significantly (*p* = 0.009) more IPE activities overall (Table 4). Similarly, age was significantly (*p* = 0.031) positively correlated with a higher proportion of IPE activities (Table 4). However, as MS progressed in their studies, the higher became the UWE-IP-D ILS by a non-statistically significant trend (*p* = 0.06; i.e. less positive attitudes toward IPE; Table 3), the less teaching activities they wished to see conducted in an IPE setting (*p* = 0.028; Table 4), and fewer hours they were willing to spend on IPE outside the fixed curriculum (*p* = 0.005; Table 5). Previous occupation in health care rendered MS less likely by a non-statistically significant trend to invest into voluntary IPE (*p* = 0.07; Table 5). Previous occupation outside health care was significantly (*p* = 0.011) positively correlated with a higher percentage of desired IPE activities (Table 4). Applying Bonferroni adjustment across 6 predictors (α = 0.008) confirmed sex (UWE-IPE-D ILS; Table 3) and medical school year (voluntary IPE extra hours; Table 5) as signifcant predictors.

**Table 3:** Estimation of the relationship between the interprofessional learning scale of the university of the West of England interprofessional questionnaire (UWE-IP-D ILS) with sex, age, medical school year, previous occupation in or outside health care and previous interprofessional education (IPE) experience by multiple linear regression analysis

UWE-IP-D ILS	B	SE	95% CI		β	t	*p*-value
			LL	UL			
Sex	2.9	1.04	0.85	4.94	0.17	2.79	**0.006**
Age	-0.19	0.18	-0.55	0.18	-0.08	-1.01	0.31
Medical school year	0.74	0.39	-0.04	1.51	0.12	1.88	0.06
Previous occupation in health care	0.31	1.14	-1.93	2.55	0.02	0.27	0.79
Previous occupation outside health care	-0.9	1.56	-3.97	2.18	-0.04	-0.57	0.57
Previous IPE experience	1.66	1.02	-0.35	3.67	0.1	1.63	0.11

Note: adjusted R^2^ = 0.03, F = 2.4, *p* < 0.05

**Table 4:** Estimation of the relationship between percentage of all teaching activities desired to be held in an interprofessional education (IPE) setting with sex, age, medical school year, previous occupation in or outside health care and previous IPE experience by multiple linear regression analysis

% IPE	B	SE	95% CI		β	t	*p*-value
			LL	UL			
Sex	-5.34	2.02	-9.32	-1.36	-0.16	-2.64	0.009
Age	0.78	0.36	0.07	1.48	0.16	2.17	0.031
Medical school year	-1.69	0.77	-3.2	-0.18	-0.14	-2.21	0.028
Previous occupation in health care	2.66	2.21	-1.7	7.02	0.08	1.2	0.23
Previous occupation outside health care	7.83	3.04	1.84	13.82	0.17	2.57	0.011
Previous IPE experience	-2.04	1.99	-5.96	1.88	-0.06	-1.03	0.31

Note: adjusted R^2^ = 0.05, F = 3.4, *p* < 0.01

**Table 5:** Estimation of the relationship between willingness to participate in voluntary extra-curricular interprofessional education (IPE) classes with sex, age, medical school year, previous occupation in or outside health care and previous IPE experience by multiple linear regression analysis

Voluntary IPE extra hours	B	SE	95% CI		β	t	*p*-value
			LL	UL			
Sex	-0.08	0.18	-0.43	0.28	-0.03	-0.42	0.68
Age	-0.02	0.03	-0.08	0.04	-0.05	-0.69	0.49
Medical school year	-0.19	0.07	-0.33	-0.06	-0.19	-2.84	0.005
Previous occupation in health care	-0.36	0.2	-0.74	0.03	-0.13	-1.83	0.07
Previous occupation outside health care	0.08	0.27	-0.45	0.61	0.02	0.3	0.76
Previous IPE experience	-0.24	0.18	-0.58	0.11	-0.09	-1.35	0.18

Note: adjusted R^2^ = 0.03, F = 2.5, *p* < 0.05

### IPE-eligible Subjects

The top three IPE-eligible preclinical subjects were Psychology / Sociology (75%), Anatomy (66%), and Physiology (55%). Psychiatry (57%), Psychosomatics (54%) and Family Medicine (52%) were marked as the three clinical subjects best compatible with IPE (Fig. 1).

## Discussion

A representative sample of MS in our private German network exhibited positive IPE attitudes (UWE-IP-D ILS mean 20.27 ± 8.16, α = 0.938), with female sex emerging as the strongest predictor of favorable scores (*p* = 0.006 post-Bonferroni) and preclinical years showing greatest enthusiasm. Over 85% supported curricular IPE integration, prioritizing Psychology / Sociology (75%), Anatomy (66%), and Clinical Mental Health / Family Medicine (> 50%)—revealing actionable priorities amid declining readiness later.

This female advantage aligns with the literature [18] as female sex emerged as a significant predictor of positive IPE attitudes – potentially explained by females’ superior communicating abilities [19] and openness to “connected learning” [20]. While Woermann et al. [14]. did not detect any sex differences in a survey of 254 German MS and 244 nursing students using the Interdisciplinary Education Perception Scale, negative arguments against voluntary IPE events were reported, primarily citing time constraints and work overload. In the same study, some MS expressed concerns about differences in competency level goals between the different professional students; a subgroup even rejected IPE fearing infringement of their own learning success [14]. Altin et al. [21] found that three-quarters of interviewees from German institutions offering continuing IPE noted limited physician engagement in such trainings, reinforcing the stereotype of doctors lacking interpersonal skills [22]. Contrary to these perceived barriers stand the findings of over 80% of our surveyed MS calling for IPE and ready to devote a mean (standard error) of 2 (± 1.41) hours per months (and some up to 5 h) to non-mandatory IPE.

Approximately half of the MS in the IRO Group had previous work experience, explaining the age effect. Yet, IPE willingness decreased across all surrogate parameters with advancing. This is consistent with Oudbier et al.‘s [18] findings and Pollard et al.‘s [17] idealism fade hypothesis. Thus, our findings add to the pivotal question of the optimal timing of IPE implementation [23], especially given the high effort for all stakeholders [24, 25]. We advocate for embedding IPE early in the curriculum to break down professional silos and promote interprofessional collaboration [26]. Accordingly, MSH launched a 2-week internal medicine / nursing internship — directly translating our preclinical enthusiasm into practice.

Previous professional experience in the health care sector unexpectedly trended negatively with IPE attitudes (*p* = 0.07) in this study, contrasting González Blum et al.‘s [27] positive findings for nursing / social work exposure. Since there was no open comment option in our survey, a solid explanation is lacking and may be multi-facetted. Interestingly, the opposite held true for those with work experience outside medicine, which is in agreement with existing literature [18]. We speculate that individuals with a medical background already feel “IPE-satisfied”. Prior negative work collaborations with physicians and other health care workers could also contribute to IPE fatigue. However, previous IPE experience as reported by 48% of the MS cohort, did not significantly factor in, which is consistent with Woermann et al.’s findings [14]. The extent to which MS were previously exposed to IPE, and whether this merely entailed serendipitous interprofessional learning (unplanned, spontaneous, and implicit learning between professional practitioners or students [28]) remains unanswered.

To complement attitude data and contribute address optimal timing of IPE timing, participants selected preferred subjects. Preclinical preferences align with prior work favoring basic sciences [11], and IPE-implementations in anatomy [25, 27, 29, 30], alongside physiology [31]. While Woermann et al. [14] endorsed IPE events on communication and ethics as well as clinical skills, we targeted core German medical curriculum subjects. Still, psychiatry and psychosomatics as well as family medicine emerged as high potential clinical areas. While our results will not drive legal changes of the upcoming curriculum changes [6], they offer empirical evidence to guide compliant curriculum design.

Next to didactic formats such as workshops, and problem-based learning [32], structured interprofessional bedside teaching rounds [33] could become viable for IPE-eligibility, complementing the many learning approaches that must be tailored to diverse learning types [34, 35].

In light of the foregoing and consistent with other authors’ impressions [36, 37] large-scale IPE implementation remains notoriously challenging. D’Amour et al. emphasize that IPE must be well embedded at the micro (teaching), meso (institutional), and macro (systemic) levels [38]. Due to uncertainties pertaining to the best IPE teaching methods [39], and the “who, what, where and when” of IPE [36], there is a risk that IPE may be poorly received by teachers and learners and ultimately prove counterproductive [40, 41]. Interprofessional cooperation with medical faculty can be difficult [13], especially at this point in time when nursing in Germany is undergoing a transition from vocation to academia amid a healthcare system heavily reliant on international migration under the Skilled Migration Act from 2020, which introduces different perspectives on professional identity [42]. We view these dynamics as an opportunity to challenge the current “intraprofessional status quo” [43] and serve as a catalyst for IPE. Kauff et al. [44] stress the value of diversity and encourage universities to embrace all students as one “health professions family”. This family at best should comprise many more collaborators such as physiotherapists, midwifery students, operating room personnel, psychologists and others.

### Limitations

A limitation of the study design is selection bias, as voluntary participation may overrepresent MS already interested in IPE. Teaching dynamics and learning environments at private medical schools may differ. Therefore, the results should not be extrapolated to other / public medical schools but can serve as a comparison. Future surveys could include additional parameters such as students’ cultural or socio-economical background, and family or parental occupations in health-related professions to better characterize participants and determine if these factors influence attitudes toward IPE. Including an open-text comment box would be worthwhile to capture missing aspects such as perceived barriers and enablers of IPE.

## Conclusion

As per the validated UWE-IP-D ILS questionnaire and expressed willingness to allocate curricular and extracurricular time to IPE, it can be concluded that the vast majority of MS from German private medical schools advocate IPE. These findings underscore the need for structured, early IPE integration in German medical curricula. Based on the results of this study, anatomy / physiology, family medicine, and medical subjects addressing mental health appear most suitable. Future research should address the role of age effects, previous employment, and prior IPE experience to better understand possible IPE-fatigue.

## Supplementary Information

Below is the link to the electronic supplementary material.

Supplementary Material 1

## Declarations

### Declaration of Generative Artificial Intelligence (AI) Usage

Perplexity AI was used to improve English grammar of the final draft beforesubmission without changing the content.
