# Supplementary material for: Attitudes Toward Interprofessional Education Among Medical Students of a German Private Medical School Network
Source: Med Sci Educ. 2026 Mar 27;36(3):1397–404. doi: 10.1007/s40670-026-02712-9 (PMC13356122; doi:10.1007/s40670-026-02712-9)
Supplement: Supplementary file 1 — Supplementary Material 1 [file 40670_2026_2712_MOESM1_ESM.docx]

# Supplementary File: Online Survey (English translation in *italics*)

# Fragebogen für Unipark

# *Unipark Survey*

Vielen Dank für Ihre Teilnahme an der Umfrage zu Ihrer Einstellung zu interprofessionellem Lernen, welches nach der WHO wie folgt definiert ist. „Interprofessionelles Lernen ist, wenn Studenten von zwei oder mehr Professionen über etwas und von und miteinander lernen, um eine gute Zusammenarbeit zu ermöglichen, die Gesundheitsresultate verbessert.“

*Thank you very much for participating in the survey on your attitudes toward interprofessional learning, which is defined by the WHO as follows: "Interprofessional education occurs when two or more professions learn about, from and with each other to enable effective collaboration and improve health outcomes."*

Welcher Universität im Hochschulverband der IRO Gruppe sind Sie zugehörig?

In which medical school of the IRO group are you enrolled?

❑ MSH, ❑ MSB, ❑ HMU Potsdam, ❑ HMU Erfurt

*❑ MSH, ❑ MSB, ❑ HMU Potsdam, ❑ HMU Erfurt*

Ihr Geschlecht ❑ weiblich ❑ männlich

*Your sex ❑ female ❑ male*

Alter: ___

*Age: ___*

Haben Sie vorgängig zu Ihrem Medizinstudium eine Ausbildung in einem Gesundheitsberuf (Krankenpflege, Physiotherapie, Osteopathie, o.ä.) absolviert? ❑ ja ❑ nein

*Did you finish any vocational training in health care (i.e. physiotherapy, osteopathy, or similar) prior to your medical studies? ❑ yes ❑ no*

Haben Sie vorgängig zu Ihrem Medizinstudium eine Ausbildung oder ein Studium in einem Nicht-Gesundheitsberuf absolviert? ❑ ja ❑ nein

*Did you finish any vocational training or studies in any other field prior to your medical studies? ❑ yes ❑ no*

Haben Sie schon eine oder mehrere Lehrveranstaltungen mit interprofessionellen Inhalten besucht? ❑ ja ❑ nein

Have you had learning activities with interprofessional content? ❑ yes ❑ no

German version of the Interprofessional Learning Scale of the University of the West of England Interprofessional Questionnaire (UWE-IP-D ILS) - 9 items

- - - - - -

In welchen der folgenden vorklinischen Fächer wäre interprofessionelles Lernen für Sie vorstellbar? Mehrfachauswahlen möglich.

*Which of the following preclinical subjects seem most suitable for you for interprofessional learning? Multiple selections allowed.*

❑ Anatomie *anatomy*

❑ Biologie *biology*

❑ Biochemie *biochemistry*

❑ Chemie *chemistry*

❑ Physiologie *physiology*

❑ Physik *physics*

❑ Psychologie / Soziologie *psychology / sociology*

❑ Terminologie *terminology*

*(Frage wird für Studenten unter dem 5. Studiensemester ausgeblendet):* In welchen der folgenden klinischen Fächer wäre interprofessionelles Lernen für Sie vorstellbar? Mehrfachauswahlen möglich.

*(This question is omitted for preclinical students):* *Which of the following clinical subjects seem most suitable for you for interprofessional learning? Multiple selections allowed.*

❑ Allgemeinmedizin *family medicine*

❑ Anästhesiologie *anesthesiology*

❑ Arbeitsmedizin und Sozialmedizin *occupational medicine*

❑ Augenheilkunde *ophthalmology*

❑ Chirurgie *surgery*

❑ Dermatologie *dermatology*

❑ Frauenheilkunde *gynecology*

❑ Hals-Nasen-Ohrenheilkunde *otorhinolaryngology*

❑ Humangenetik *genetics*

❑ Mikrobiologie *microbiology*

❑ Innere Medizin *internal medicine*

❑ Kinderheilkunde *pediatrics*

❑ Klinische Chemie *clinical chemistry*

❑ Neurologie *neurology*

❑ Orthopädie *orthopedics*

❑ Pathologie *pathology*

❑ Pharmakologie *pharmacology*

❑ Psychiatrie *psychiatry*

❑ Psychosomatische Medizin *psychosomatics*

❑ Rechtsmedizin *forensic medicine*

❑ Urologie *urology*

Wieviel % der Lehre sollten Lehrveranstaltungen mit interprofessionellem Lernen sein?

❑ 0 – 100%

How much percentage of all teaching activities would you allocate to interprofessional learning?

*❑ 0 – 100%*

Wieviele Stunden pro Monat wären Sie bereit, in Lehrveranstaltungen mit interprofessionellem Lernen zusätzlich (also extra-curriculär) zum aktuell laufenden Semester zu investieren? ____

*How many additional hours per month (i.e. extra-curricular) would you be willing to invest into interprofessional teaching activities during the current semester? ____*
